# Beyond noise to function: reframing the global brain activity and its dynamic topography

**DOI:** 10.1038/s42003-022-04297-6

**Published:** 2022-12-08

**Authors:** Jianfeng Zhang, Georg Northoff

**Affiliations:** 1 Center for Brain Disorders and Cognitive Sciences, Shenzhen University, Shenzhen, China; 2 School of Psychology, Shenzhen University, Shenzhen, China; 3 Mental Health Center, Zhejiang University School of Medicine, Hangzhou, China; 4 Institute of Mental Health Research, University of Ottawa, Ottawa, Canada; 5 Center for Cognition and Brain Disorders, Hangzhou Normal University, Hangzhou, China

**Keywords:** Consciousness, Dynamical systems, Cognitive neuroscience

## Abstract

How global and local activity interact with each other is a common question in complex systems like climate and economy. Analogously, the brain too displays ‘global’ activity that interacts with local-regional activity and modulates behavior. The brain’s global activity, investigated as global signal in fMRI, so far, has mainly been conceived as non-neuronal noise. We here review the findings from healthy and clinical populations to demonstrate the neural basis and functions of global signal to brain and behavior. We show that global signal (i) is closely coupled with physiological signals and modulates the arousal level; and (ii) organizes an elaborated dynamic topography and coordinates the different forms of cognition. We also postulate a Dual-Layer Model including both background and surface layers. Together, the latest evidence strongly suggests the need to go beyond the view of global signal as noise by embracing a dual-layer model with background and surface layer.

## Abstract (web summary)

Zhang and Northoff revisit the findings of global signal in fMRI from healthy and clinical populations and suggest that a dual-layer model of global signal, which takes into account both background and surface layer, may be more accurate than a single-layer view

## Introduction

The relationship between global and local activity changes is a common phenomenon in the natural world which, among other examples of complex systems, can be observed in climate change and economy. Global warming of the earth atmosphere affects the climate in different countries and continents in different ways depending on their respective local-regional features (like ice melting in colder regions but the deserts wetting in warmer regions)^1,2^. Similarly, the global economy strongly affects economies in different countries albeit in different ways depending, among other factors, on the level of their development^3,4^. What holds for climate and economy may also apply to the brain as another complex system in a more or less analogous way.

Recent evidence suggests that, just like in the cases of economy and climate, the brain too displays ‘global’ activity (see below for defining the term ‘global’) that modulates and is represented non-uniformly across various local regions and networks. That may, in part, be related to subcortical-cortical modulation: subcortical nuclei like serotoninergic raphe nucleus, acetylcholinergic nucleus basalis meynert, and dopaminergic substantia nigra modulate cortical activity in a multiregional ‘global’ way including the balances between different networks^5–7^. Additionally, recent studies in animals show that multiple regions are implicated in inducing and mediating one specific behavior^8^—this supports the potential role of the brain’s more global activity in behavior.

The apparent importance of ‘global’ activity for brain and behavior stands in contrast to its measurement in human fMRI, though. ‘Global’ activity is measured by the global signal (GS) in fMRI^9–11^. When speaking of GS, fMRI researchers defined it operationally as the average of whole brain voxels^10–16^ or voxel within gray matters^17–22^, as empirically they are highly correlated^10^. The fMRI researchers are first confronted with a predominantly methodological connotation^9,12,16^. Inclusion or exclusion/regression of GS in fMRI data significantly impacts relationship between task positive and negative networks^9,13–15,23,24^. For instance, regression of GS may introduce anti-correlation of these networks which otherwise, in the presence of GS, may no longer stand in a negative relationship^9,10,13,16,25–29^. Additionally, GS has been associated with extra-neuronal sources^30^ like respiration^10,16,31,32^, heartbeat^33,34^, and blood transit effect^35–38^. Together, these observations support a primarily negative view of GS culminating in the need for its regression and elimination from the data^10^.

**Fig. 1:**
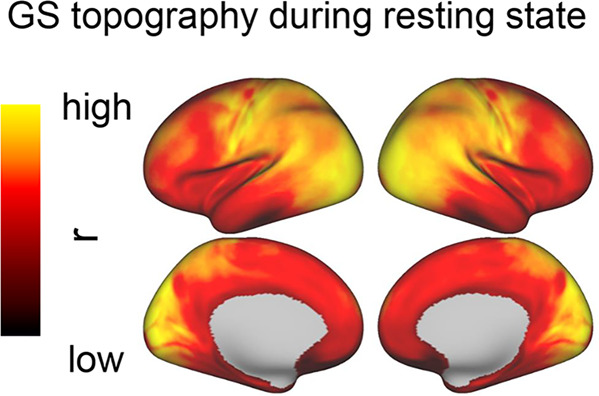
GS topography during resting state. A reliable relationship between GS and cortical regions has been observed across various studies^10,17,19–22,41–44^. In general, the primary sensory regions (i.e., sensorimotor and visual cortex) show higher correlations with GS (as indicated by the more yellow color), and the higher-order cortical regions show low correlations with GS (as indicated by the red color).

However, recent studies combining ECoG/electrophysiology and fMRI demonstrate a direct relationship of fMRI-based GS with electrophysiological measures; these findings suggest that GS is not merely non-neuronal noise but also an important source of neuronal activity itself^39,40^. Furthermore, various studies show that GS is represented reliably in different degrees in different regions, i.e., it displays a dynamic topography (Fig. 1)^10,17,19–22,41–44^ (see Box 1 for different ways of calculating the spatial pattern, in terms of GS topography). The potential behavioral and cognitive relevance of GS topography is supported by the observation of topographical changes in various neurologic and psychiatric disorders as these show major alterations in perception and cognition (see below for details). Together, these findings suggest that GS may take on a yet to be defined physiological role and function in both brain and behavior.

Reviewing recent findings, the goal of our review is to go beyond GS as mere noise^9,10,16^, and illustrating its physiological and neuronal relevance. For that purpose, we highlight the potential role and function of GS and its spatial representation (i.e., GS topography) for both brain and behavior. Specifically, we demonstrate that GS displays a specific physiological basis as it is closely coupled to bodily signals like respiration through subcortical-cortical infraslow phase-based mechanisms which, psychophysiologically, mediates the level of arousal. At the same time, GS coordinates the cortical regions’ and networks’ activities in a dynamic-topographic way which organizes and structures different forms of cognition. Just as in other complex systems like climate and economy where global effects non-uniformly impact local-regional changes, we, based on these recent findings, postulate a dual-layer model (DLM) of GS with both background and surface layer: the more global background activity operating as infraslow waves^45,46^ structures and coordinates the more local-regional surface activity in a dynamic-topographic way through, in part, phase-based mechanisms. Such dual-layer model sheds a novel light on the physiological role of GS in brain–body coupling and how that, in turn, mediates the brain’s dynamic topography including its relation to behavior and cognition.

Box 1 Different measures of GS topography **GS correlation (GSCORR):** GS correlation (GSCORR) is the most widely accepted measure for GS topography. GSCORR is calculated by using Pearson’s correlation between GS (the averaged time course across gray matters) with the time series in each voxel. By using the GSCORR, a typical GS topography has been observed in healthy controls^10,21^ as well as alterations in GS topography in psychiatric disorders^22,44,91^ and loss of consciousness in disorders of consciousness^20^. Additionally, besides abnormal GS topography, changes in the temporal dynamics of GSCORR have also been calculated by the time lag between GS and local activity as demonstrated in acute stroke (and related to a perfusion deficit)^103^.

**Global functional connectivity/global brain connectivity (GFC/GBC):** Another widely used measure for GS topography is the global functional connectivity (GFC), or in some studies called as global brain connectivity (GBC)^22,104–109^. The GS topography obtained by GFC/GBC is measured as the averaged Pearson’s correlation between one voxel with the other voxel, which differs from the GSCORR as for GFC/GBC, it is the r values that are averaged whereas for GSCORR, it is the time series that is averaged. Despite their difference in calculation, the obtained spatial patterns by GFC/GBC and GSCORR are almost identical, especially if the time series is normalized (z-score)^22^. GFC is also widely used for cognitive^110^ and clinical studies^22,87,106–109,111^ which are not always subsumed under the topic of GS but rather under the term of functional connectivity in general. The disadvantages of GFC are that it is computationally more expensive, and cannot display temporal information (e.g., the time lag) between global and local activity^103^. However, GFC has the advantage that it allows performing GS regression^90,109,110^ in case one regards GS as noise. In contrast to GFC, GS regression cannot be performed in GSCORR, as this approach will methodologically lead to GSCORR close to zero. The influence of GS regression on GFC remains an open issue. Specifically, it is not clear whether the elimination of GS removes the neuronal-informative or extra-neuronal noisy parts of GFC^112^, as well as whether the spatial pattern of GS topography still persists after GS regression.

**GS regression (GSR):** GSR was first introduced as a methodological issue to investigate how the result of functional connectivity is impacted by the regression of GS^9,13,15,25^. In addition to taking GSR as a confounder, recent studies further investigated the spatial pattern of beta weights by using GS as regressor in cognitive and clinical studies^17,19,111^. The topography obtained by the beta weights of GS also mimics the pattern observed by other measures like GSCORR, GFC, and CAPs at the GS peak, suggesting the robustness of GS topography across different measures. However, to our knowledge, no study has directly compared the weights from GS regression with other measures, which should be worth doing as they may be influenced differently by the phase and amplitude of local activity^22^.

**Co-activation patterns (CAPs):** All the three measures above account for the relationship between local and global activity across time. The co-activation patterns (CAPs) was initially developed to identify time-varying default mode network profiles based on a few frames with suprathreshold signal of posterior cingulate cortex (PCC)^113^. Recent findings demonstrate that the GS topography can also be traced to instantaneous recurring dynamic CAPs at the peak of the GS^21,41^. In other words, the GS topography measured by functional connectivity is mimicked by the whole brain’s spatial pattern across networks at the peak time point of the GS, i.e., GS peak^21^. The GS topography observed by CAPs is not an independent spatiotemporal unit but is rather dynamic as it changes across time in dependence on the ongoing phase of GS fluctuations; therefore, GS topography can be further decomposed into a subset of distinct CAPs^21,44^.

**Temporal independent components analysis (tICA)**: A major concern of GS and its topography is whether it represents signal or noise. Recently, Glasser et al.^78^ applied the temporal independent components analysis (tICA), to decompose the GS into a set of global structured signal, with characterized spatial patterns. For investigating the temporal dynamics of each global fluctuating components, tICA can be a useful tool in understanding the functional relevance of GS topography by identifying the exact functionally relevant components. As this approach is relatively new in global signal analyses, future studies are warranted to further validate the distinction of noisy and neural parts of global activity^114,115^.

**Quasi-periodic pattern (QPP):** QPP is a pseudo-periodic spatiotemporal pattern observed at a time scale around 20 s^116^. The QPP is calculated by a correlation-based iterative approach^117^. As showed by Yousefi et al.^118^, one QPP strongly correlates with global signal, and its spatial pattern mimics the global signal topography. More importantly, the regression of slow respiratory and cardiac induced signal fluctuations reduces the global signal related QPP, and make the other QPP, i.e., the one with anti-correlation between DMN and TPN, become stronger.

**Systemic low-frequency oscillations:** The global signal may originate from systemic circulatory oxygenation fluctuations in the periphery^36,38,119^. This low-frequency peripheral oscillation tracks the global signal and the global signal topography may indicate differential blood transit time in the cerebral vasculature. Potential sources of this systemic circulatory effect include vasomotion, fluctuations in arterial CO_2_ and/or Mayer waves. Functionally, this systemic low-frequency oscillation may associate with the fluctuation of arousal^45^.

**Complex principal component analysis (CPCA):** CPCA is a complex-valued extension of a popular dimension reduction technique. Using CPCA, Bolt et al.^46^ shows that the brain can be characterized into three main components, and these components could explain the spatiotemporal dynamics illustrated in previous findings. They show that pattern one is strongly correlated with the global signal. The time course of pattern one and the global mean time course are statistically indistinguishable. And the global signal topography also shows a high similarity with the map of pattern one.

**Lag threads: “**Lag threads”, also called as lag projections^46^, describes the temporal sequences of propagated activity in the brain at the time scale around 2 second^120,121^. Lag threads are computed from the average pair-wise time delays between BOLD time courses and represent the average ‘ordering’ in time of BOLD amplitude peaks across the brain. The lag threads map shows a high spatial correlation with the pattern of global signal related component in complex principal component analysis^46^.

## Neural and physiological basis of the GS

### Neural correlates of GS

Given that our scope is to review the function of GS, a fundamental and prerequisite question is whether this infra-slow ‘global’ activity has a physiological/neural basis. Reviewing several studies combining GS in fMRI with electrophysiological measurements in mainly monkeys^39–41,47–51^ and humans^40^, one key electrophysiological feature is that GS exhibits different relations to the band limited power of different frequency ranges. For instance, infraslow frequency ranges (<0.1 Hz) show a much higher relationship, i.e., correlation with GS than faster frequencies like those in the slower (0.1–1 Hz), and faster ranges (1–100 Hz)^39,47,50^. In addition, the broadband power fluctuation, rather than oscillatory (i.e., alpha) power fluctuation, in EEG also demonstrate a strong relationship with global signal in fMRI^40,51^. These results suggest that GS is strongly driven by the long cycle durations of the infraslow frequency fluctuations, and therefore may provide a slow temporal structure that organizes the activity of faster frequencies through phase-amplitude coupling. This points to a special role of infraslow frequency range for GS as distinct from the one of faster frequencies (whose contribution to GS remains to be established). In addition to the frequency range, the degree of spatial extension or distance may be an important factor. Several studies show that slower delta/theta (1–8 Hz) activity and faster gamma power (40–80 Hz) contribute strongly to the spatial extension of neural activity beyond single regions on the cortical level and subsequently to GS^40,41,47^. In contrast, the alpha/beta range (10–30 Hz) is not related to such global extension but remains rather local as restricted to specific regions like visual/posterior cortex and thalamus and consequently show low degrees of contributions to GS^39–41,49^.

Together, these data suggest a distinct electrophysiological basis of GS at the infraslow frequency range, which, as recent studies show^45,46^, may manifest in so-called “standing and traveling waves”, where standing waves refer to stationary oscillations exhibiting no time-lagged statistical dependencies across space, and traveling waves refer to oscillations in a spatial field with non-zero time-lag statistical dependence across space (see details in Bolt et al.^46^). These infraslow fluctuations are (i) spatially extended in a more or less global way (with ‘global’ being understood in a relative way in terms of degree of spatial extension as distinct from an absolute way as involving all and every brain region); and (ii) temporally related to faster frequency ranges. Accordingly, GS can physiologically be characterized by an infraslow dynamic topography operating in a global way across more or less the whole brain including both subcortical and cortical regions.

### Physiological correlates of GS

The findings above suggest that the global signal is associated with a widespread infraslow modulation of neural activity. Where does this global signal originate? Given that the connection between neural activity and fMRI-based BOLD signal is through neuro-vascular coupling, previous studies have investigated whether the global signal is associated with global metabolic change, e.g., the physiological sources of global signal^35–38,52,53^. Indirect indices of metabolic change are respiration and cardiac activity; these are indeed associated with the global signal, as the modeled global signal by respiration and cardiac activity can explain a large portion of the empirical global signal^34,53^. It shall be noted that respiration itself (as well as other physiological signals like cardiac activity and cerebral vasomotion) exhibit their own fluctuations in the infraslow frequency range which correlate highly with the ones of the brain’s global signal^35–38^. This is, for instance, supported by Yao et al.^37^ who observed that arterial blood flow^37^ predicts the global signal with consistent time delays. In sum, these findings illustrate close relationships of the global signal with body-based physiological fluctuations related to the temporal dynamics of metabolic consumption.

We shall note that these physiological sources of the global signal do not suggest that the GS is an exclusively non-neuronal noise. On the contrary, recent findings rather demonstrate that these physiological fluctuations are coupled with the specific spatiotemporal dynamics of the brain’s global neural activity^52,54^. Given the high correlation of physiological and neural signals in their infraslow fluctuation dynamic, one may tentatively assume that the latter’s long cycle durations may be key in integrating and synchronizing the two kinds of signals in a temporal way, e.g., through their corresponding timescales and/or phase cycles^55,56^. The temporal features of both physiological and neural signals may thus be shared as their “common currency”^57,58^—the shared dynamic may enable their direct communication across the physical boundaries of brain and body through for instance phase-based synchronization. Finally, it shall be pointed out that such coupling of physiological signals and global neuronal activity, e.g., brain–body coupling, seems to carry important psychophysiological functions as it mediates the level of arousal^45^ as well as cognitive relevance as it mediates trial-by-trial behavioral performance^59^. The exact neuronal mechanisms of such brain–body coupling through GS including its relationship to behavior and cognition remain to be explored, though^35,36^.

## Function of GS I—mediating the level of arousal

### Neurophysiological evidence linking the GS to the level of arousal

The term of arousal has been defined and understood in many different ways. According to the studies we mentioned below, the concept of arousal here is conventionally defined as a transient intrusion of being awake into unconscious states like sleep or anesthesia^41,49,60,61^, or a temporary alteration of the vigilance/alertness level^59^. Quantitatively, the level of arousal can be measured by clinical scales^60,62^, behavioral index (e.g., pupillometry)^49^, EEG spectrum (e.g., alpha power, alpha/theta ratio or sequential spectral transitions, SST)^41,49,59,63^ or fMRI arousal spatiotemporal markers^41,59,64–66^.

Various lines of evidence in linking GS fluctuation to the level of arousal have been observed^39,41,49,61,67^. Exogenously, the level of GS is associated with the level of arousal altered by caffeine^26^ or pharmacological drugs as in anesthesia^20^. While endogenously, the level of GS is modulated by internally-oriented factors like sleep^20,63^, circadian rhythms^18^, or temporal variation of alertness^59^.

How are the different types of arousal indices related to GS? Chang et al.^49^ took the monkeys’ changes from eyes closed to eyes open across time as index of behavioral arousal. They also investigate the same monkeys in fMRI where they observe a widespread negative correlation with behavioral arousal. Subsequently, a so-called ‘fMRI arousal index’ is generated by correlating the widespread arousal pattern with instantaneous co-activation pattern. Both arousal indices, fMRI and behavioral, are then correlated with each other. This yields highly significant correlation of behavioral and fMRI arousal indices: fluctuations in the behavioral arousal index are related to corresponding fluctuations in the fMRI arousal index.

The spatial pattern of the fMRI arousal index is also confirmed in human beings by its similarity to an instantaneous co-activation pattern that is phase-locked to the peak of GS—that suggests a key role for phase-related mechanisms in mediating the impact of GS on arousal^41^ (see also below). Furthermore, as an index of the level of vigilance and arousal, the occurrence of this co-activation pattern predicts the behavioral response variability^59^. Yet another recent study observed that global phase-related fluctuations, e.g., traveling waves are related to the fluctuations in the level of arousal^45^. Finally, in order to provide an electrophysiological basis of the fMRI index of arousal, they also obtained simultaneous ECoG measuring the beta- and theta-range power index. The fMRI index of arousal correlates significantly with the beta- and theta-range power index (15–25 Hz and 3–7 Hz, respectively), suggesting that the widespread co-activation pattern has a distinct electrophysiological basis in the power spectrum^41^.

How do the physiological contributions of GS relate to arousal? By including fMRI resting state, physiology (i.e., respiration), and electroencephalogram (EEG, alpha power), Yuan et al.^68^ observed that, using resting-state fMRI-EEG, the degree of respiration correlates with EEG alpha power serves as an index of the level of arousal/vigilance. Additionally, trial-by-trial behavioral performance in reaction time was also related to the “physiological networks”^53,59^, which refers to the brain regions correlating with the activity of systemic physiology (i.e., respiration and heartbeat). Finally, recently findings suggested that respiration drives the fluctuations of arousal, and, through the phase-based synchronization, couples with the spatiotemporal dynamics of brain networks^45^. Taken together, these findings suggest an intimate relationship of infraslow fluctuation of respiration with the dynamics of GS, which in turn mediates the level of arousal. Infraslow dynamic thus seems to be shared by physiological (respiration), neural (GS), and basic psychological (arousal) signals serving as their “common currency”^57,58^. Thereby, the GS might be viewed as a hybrid neuronal-vascular-physiological signal; and it is exactly this feature that makes it possible for GS to mediate the level of arousal thus accounting for the tight coupling of GS and arousal level.

### Subcortical-cortical modulation of GS mediates the level of arousal

The close relationship between GS and arousal is further supported by arousal-related subcortical-cortical modulation. Anatomically, the inputs of respiration and cardiac activity, which highly correlate with GS, are processed in subcortical nuclei and subcortical-cortical connection as these are implicated in arousal modulation. The respiration pattern can, for instance, be modulated by neural activity in the locus coeruleus, a vigilance center^69^. Correspondingly, the relationship of arousal and GS is further supported by various lines of evidence from subcortical-cortical modulations.

The empirical findings suggest that subcortical regions related to arousal may be suitable candidates for the origin of arousal-related GS fluctuations^41,48^. Liu et al.^41^ demonstrate that subcortical activity exhibits correlation with cortical GS peak albeit in a negative way opposite to cortical regions: the troughs of subcortical activity fluctuations correlate with cortical GS peaks which, in turn, correlate in a positive way with activity peaks at the cortical level. These data suggest that GS is related to both subcortical-cortical and cortical-cortical modulation.

The subcortical regions correlating negatively with cortical GS peak include the thalamus (dorsomedial), the basal forebrain, and midbrain (above pons, may be substantia nigra)—they all show decreased signals during cortical GS peaks^41^. The strongest subcortical decrease is observed in the basal forebrain, the Nucleus Basalis Meynert (NBM) that contains acetylcholine which is known to modulate the arousal level. The key role of the NBM in mediating arousal and GS is further supported in a subsequent monkey study where NBM lesion causes changes in both arousal level and GS^48^. Even though subcortical NBM lesion causes GS decrease on the cortical level, the cortical typical resting-state network topography is maintained. This suggests that subcortical NBM and acetylcholine selectively modulate cortical GS in a truly global somewhat coarse-grained way as distinguished from more specific fine-grained topographical effects in specific regions or networks of the cortex. Taken together, these findings strongly support a role of GS in mediating the level of arousal as driven by subcortical regions and their apparent anti-correlation with cortical GS.

### GS and arousal in states—evidence from disorders of consciousness

Various studies during anesthesia in both human^20,60,70–72^ and animal^20,73,74^ as well as unresponsive wakefulness syndrome (UWS) in human^72^ suggest that the brain’s GS is strongly reduced if not absent in these states associated with low arousal level. These findings further support the assumption that the level of GS is central for maintaining the level of arousal as the most basic dimension of consciousness (Northoff and Lamme^75^ for a review of the different theories of consciousness).

This assumption is tested in a recent study by Tanabe et al.^20^ Tanabe et al.^20^ conducted fMRI in a variety of different groups including both animal (rat) and human anesthesia with different propofol dosages (high, medium, low) in rats and different levels (wakefulness, sedation, and anesthesia) in humans. In addition, they include human subjects suffering from minimally conscious state (MCS) and UWS as well as subjects in different sleep stages (N1-3).

They measure the amplitude of GS, as well as the functional connectivity of the GS to all single voxel/regions in the brain. Both the amplitude and functional connectivity of GS exhibit major reductions in complete anesthesia in both rats and humans as well as in N3 sleep and UWS. While the intermediate stages like sedation, medium propofol dosage, N1/N2, and MCS show intermediate levels of amplitude and functional connectivity of GS as they are higher than during the complete of unconsciousness and lower than in the fully awake state. This further suggests that the level of GS may correspond to the level of arousal as the most basic dimension of consciousness; this, as the data, seems to hold across the different conditions and their distinct neuronal origins. That suggests a most basic and fundamental role for GS in arousal prior to and beyond the lesions or changes in particular regions or networks (as manifest in the different kinds of disorders of consciousness).

In sum, GS displays a distinct electrophysiological basis and mediates the fluctuations in the level of arousal by its own fluctuations on physiological, subcortical and cortical levels. Initial evidence in humans suggests that subcortical-cortical GS, through its coupling to the body’s physiological signals, is key for maintaining arousal as manifest in the state or level of consciousness. Together, this suggests that GS operates as subcortical-cortical infraslow background right at the interface of neural and physiological signals, e.g., brain and body. Such brain–body coupling, by modulating the global metabolic-energetic level for neural system, in turn, provides a neural predisposition (rather than a neural correlate)^75–77^ for the level of arousal, i.e., level or state of consciousness, as basis for our most basic behavioral navigation within the environment.

## Function of GS II—coordinating different forms of cognition

Mediating the level of arousal indicates that GS functions by regulating the brain state in a most basic and general manner. Does GS also mediate cognition and associated behavior in a more specific way? Following various lines of findings from both healthy and psychiatric groups, we suppose the second function of GS to consist in coordinating the different forms of cognition and their related behavior through the spatial relationships and patterns of networks/regions at the cortical level, i.e., GS topography. For that purpose, we review two lines of evidence: the GS topography during different cognitive states in healthy subjects, as well as the differential changes in GS topography in various psychiatric disorders.

### GS coordinates rest and task states as manifested in GS topography

Although the GS is distributed over all the whole cortex across the gray matter, recent studies demonstrate a non-uniform topographical distribution of GS across brain regions in both monkeys^48^ and humans^10,17,19,21,41^ (Fig. 1). Such topographical distribution of GS is observed already during the resting state: primary sensory (visual and auditory cortex) and sensorimotor cortex exhibit high levels of GS during the resting state whereas GS is lower in higher-order cortical regions including the prefrontal cortex.

How is GS related to cognition? Since GS is investigated mainly as a methodological issue of resting state^9,10,13,15,16^, the issue about whether and how the GS may mediate cognitive functions is just at the initial stage. A recent study in resting state indeed suggests that the GS topography correlates with behavior^17^. By decomposing global signal into subcomponents via temporal Independent Component analysis (tICA), Glasser et al.^78^ observed task-related components contained in the GS suggesting the potential relevance of GS for task states and related cognition.

In another study, Zhang et al.^21^ demonstrated that, as distinct from rest, different tasks were associated with distinct patterns in GS topography. Most notable, the transition from rest to task states (as well as the transition between different task states) could be traced to changes in occurrence rate of the co-activation patterns at the peak of GS. Together, these findings suggest close relationship between different neural states (like rest and task as well as different task states) and different patterns of GS topography.

### GS coordinates internal physiological signals and external task demands

Earlier findings in resting state demonstrate that the GS topography mimics the spatial pattern of respiration effects^31,32^ where the predominant areas are the sensorimotor cortex, suggesting the high relevance of physiological signals (e.g., respiration) for GS topography^10,16^. By convolving the temporal response functions with respiration and heart-beat, the spatial pattern of physiological response function also mirrors the topography of GS. This further suggests that physiological signals like respiration may indeed provide an extra-neuronal source of GS^34,53^, or may relate to the interoceptive processing which is dominated during resting state but can be shaped by extra exteroceptive processing during task states as mentioned above.

By comparing the temporal course of GS fluctuation with the temporal course of the respiration-related fluctuation, Zhang et al.^21^ observed that their relationship changes during the transition from rest to task states: their correlation is higher during rest and decreases in a task-unspecific way during the different tasks. If GS in sensorimotor cortex were reflecting nothing but the respiration-related inputs, one would assume that it should remain the same during both rest and task states. Instead, sensorimotor GS may not only be modulated by the interoceptive inputs like respiration, as predominant during rest, but also by exteroceptive inputs during task states, probably indicating a competition between intero- and exteroceptive processing.

Together, these findings tentatively suggest that GS cannot be identified completely with physiological signals like respiration fluctuation. Instead, GS seems to exhibit more of a coordinating or integrating function for intero- and exteroceptive inputs: it may mediate their continuously changing balances as for instance during the transition from rest to task states as manifested in the changes of GS topography.

### GS mediates cognitive changes in psychiatric disorders

How does the GS topography link with the behavior and cognition? Another line of evidence that indirectly supports function of GS as coordinating different forms of cognition comes from psychiatric disorders^79,80^. These disorders all show changes in their internally-oriented cognition (like self, mental time travel or mind-wandering) relative to their externally-oriented cognition. Given that internally-oriented cognition is already present in the resting state, changes in the latter’s GS topography may be mediate the former. Therefore, in this part, we will describe resting-state evidence of GS changes in various psychiatric disorders like schizophrenia, bipolar disorder, major depressive disorders and others.

#### Abnormal GS and its topography in schizophrenia

Schizophrenia is characterized by changes in both GS and its topography. Yang et al.^81^ first observe significantly higher levels of GS across the whole brain in two schizophrenia samples. In addition to the level of GS, in a later study from the same group, topographical differences are also observed in schizophrenia. Yang, et al.^19^ report significant GS representation decreases in sensorimotor networks in schizophrenia while it is increased in higher-order association networks. Further, lower-order sensorimotor and higher-order association networks’ GS anti-correlate in healthy subjects which is highly diminished in schizophrenia. In another study, Wang, et al.^44^ demonstrate that this topography can be subdivided into different states whose dynamic alternations in sub-states were correlated with clinical scales.

However, findings are not fully consistent in schizophrenia. Argyelan et al.^82^ and Argyelan et al.^83^ reported decreased (rather than increased) global functional connectivity in unmedicated schizophrenic patients which also correlates with their decreased processing speed in cognitive tasks (see Hahamy et al.^84^ for similar findings of GS reduction in schizophrenia). The inconsistences may relate to different approaches in measuring GS topography, as Yang et al.^19^ used beta value in GS regression while the other studies employed global brain connectivity (see details for measures in Box 1) or different weights of non-neuronal noise in GS.

Together, these findings demonstrate that schizophrenia exhibits abnormalities in both GS and its topography in lower-order sensory and higher-order cognitive regions. Abnormal GS topography, in turn, may contribute to the various perceptual and cognitive behavioral abnormalities like the confusion of internally- and externally-oriented cognition as it is typical for schizophrenic symptoms like delusion, thought disorder, passivity phenomena, auditory hallucination, and ego-disturbances^55,79,85^.

#### Abnormal GS topography in other psychiatric disorders

**Fig. 2:**
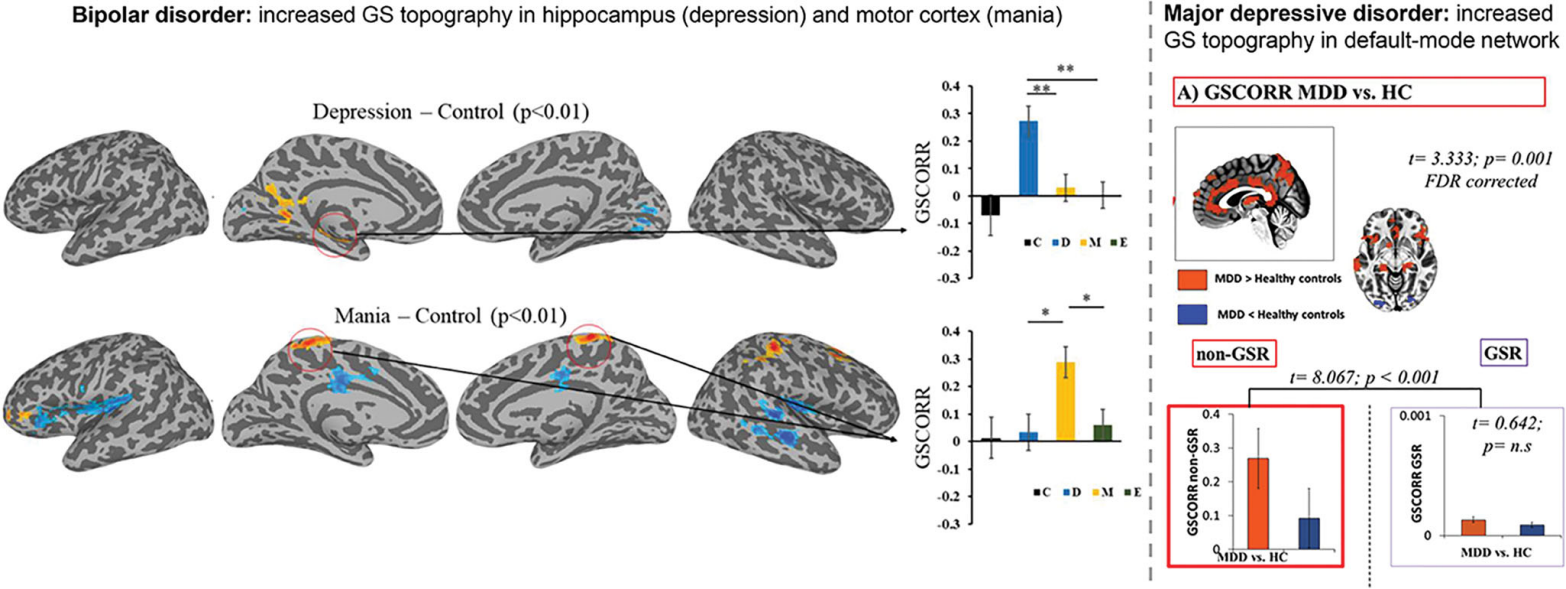
Altered GS topography in different psychiatric disorders. The GS topography is significantly altered in the different phases of bipolar disorder, with increased GSCORR in hippocampus (and parahippocampus/fusiform gyrus) in bipolar depression and motor cortex in bipolar mania (from Zhang et al.^22^). In major depressive disorder, the GS topography is increased in default-mode regions that shows abnormally strong global functional connectivity with all other regions, i.e., non-DMN in the rest of the brain (from Scalabrini et al.^89^). GS Global signal, GSCORR Global signal correlation, C Control group, D Depression, M Mania, E Euthymic, MDD Major depressive disorder, HC Healthy control, GSR Global signal regression.

Unlike elevated GS level in schizophrenia, findings in bipolar disorder (BD) show normal levels of GS^22,83^. However, GS topography is abnormal in these patients. Zhang et al.^22^ show increased GS representation in motor cortex in mania which, most likely, is related to their increased motor activity, i.e., psychomotor agitation. While in depressed BD the hippocampus exhibits increased GS as possibly related to the increased autobiography memory recall in these patients. Hence, abnormal shifts in GS topography may be related to corresponding shifts or dysbalances in behavior and cognition as in motor activity and memory recall (Fig. 2).

One may assume that these subjects’ resting state activity in hippocampus (depressive BD) and motor cortex (manic BD) may display elevated GS-based activity levels in rest that are “normally” only observed in task states (like during motor or autobiographical memory recall tasks, see Zhang et al.^22^ for such suggestion). However, the assumption of such “virtual” task-like states being already present during the resting state needs to be investigated in future studies probing real tasks; if the hypothesis is correct, one would expect decreased task-related activity and smaller or no rest-task differences (see Golesorkhi et al.^55^ as well as Northoff et al.^86^ for support of such reduced rest-task differences in schizophrenia).

Yet another condition is major depressive disorder (MDD). Clinically, MDD is characterized by increased internally-oriented cognition like mind wandering, i.e., rumination, and self-referential thought which are typically associated with increased regional/network activity in default-mode network (DMN). Various fMRI findings observe abnormal GS correlation to the regions in DMN regions like medial prefrontal cortex and hippocampus during resting state^87–92^ and task-related activity^93^, which correlates with depressive symptoms^91^ and predicts treatment response^92^.

Most recently, Scalabrini et al.^89^ demonstrate that abnormal within-DMN FC is related to alterations in GS topography. Specifically, the FC of non-DMN networks to the DMN is significantly higher in MDD than in healthy subjects 373. Moreover, the degree of DMN-non-DMN FC, i.e., the abnormal GS topography, could predict clinical diagnosis to a high degree, i.e., 90%, as revealed in vector machine learning. Together, these findings suggest abnormal global-to-local shift of GS topography towards DMN where GS is represented in increased degrees at the expense of its representation in non-DMN (Fig. 2).

Following the neuronal shift of GS towards the DMN, the behavior in MDD may also shift from non-DMN related externally-oriented cognition to abnormally strong representation of internally-oriented cognition—this is exactly what can be observed in symptoms like increased mind wandering, i.e., rumination, and self-referential thought, i.e., increased self-focus^80,94^. We therefore hypothesize that abnormal GS topography with its abnormally increased shift from non-DMN to DMN may be closely related to the abnormal shift towards internally-oriented cognition, i.e., increased self-focus, mind-wandering, and autobiographical memory retrieval, at the expense of externally-oriented cognition, i.e., decreased environment-focus with decreased perception^79,80^.

### Different interpretations of GS topography

**Fig. 3:**
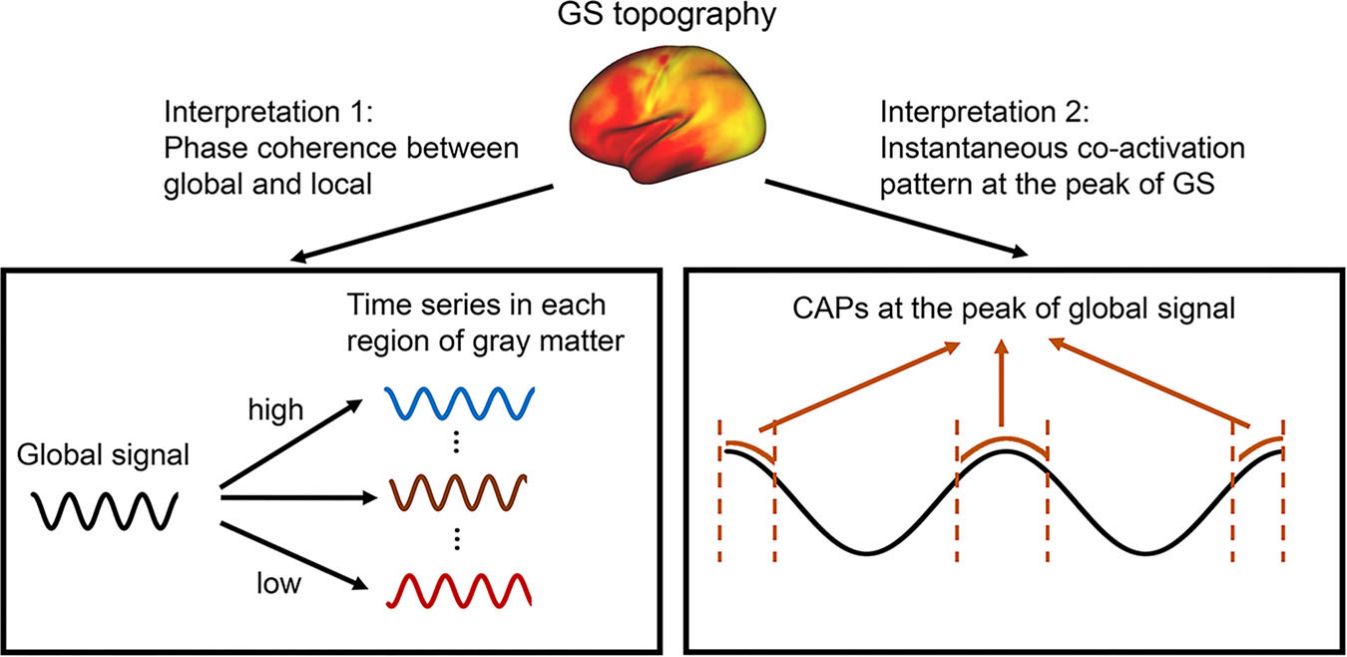
Two interpretations of GS topography. Interpretation 1 suggests that the GS topography is the degree of the phase coherence between global and local activity. Interpretation 2 suggests that the GS topography is constituted by the co-activation patterns (CAPs) of different networks as being phase-locked to the peak of GS, i.e., the instantaneous CAPs with zero-phase lag to GS. Global signal GS; Co-activation patterns CAPs.

What is the underlying interpretation of the GS topography and its transition across different states in healthy and patient groups? The question is not fully resolved yet. We here describe two possible interpretations as reported in various studies (Fig. 3). The first interpretation of GS topography may be based on the level of phase coherence between global and local activity. GS, as defined by its measure, is the sum of local activities across gray matter. As a consequence, taken in a mathematical sense, GS can be driven by the amplitude of the activity within local regions or, alternatively, by the relationship between regions, i.e., their phase coherence. By comparing the spatial topography between GS correlation (GSCORR), global functional connectivity (GFC) (determined only by phase coherence) and intra-regional amplitude (as calculated by neural variability with the standard deviation), Zhang et al.^22^ demonstrate that GS topography mainly is related to the level of phase coherence between different regions rather than the amplitude, i.e., the simple addition all regions’ amplitude (see Box 1 for the different measures of GS topography).

Note that we here only discuss the phase-based or amplitude-based features of GS while leaving out cross-frequency phase-amplitude interaction/coupling related to GS: the fMRI signal is a rather narrowband signal which limits the possibility for investigating the cross-frequency coupling including phase-amplitude or amplitude-amplitude coupling. Neuro-electrophysiological findings do indeed support that GS may be related to phase-amplitude coupling between infraslow phase (<0.1 Hz) and high frequency amplitude^51^. However, empirical evidence of linking such cross-frequency coupling to the level of arousal and cognition is still missing. MEG studies providing reference-free measurement and high spatial resolution, could provide important insights into cross-frequency coupling as potential neural correlates of fMRI GS and its topography including its infraslow phase-dependent cognitive and behavioral changes in the future.

The second interpretation of GS topography focuses on instantaneous co-activation pattern (CAPs) that occurs at a specific phase of GS (i.e., the peak of GS)^21,41,95^. This hypothesized mechanism suggests that the GS topography is not a static pattern describing the relationship between global and local, but a dynamic pattern describing that the instantaneous CAPs are phase-locked to the peak (rather than the trough) of GS. This suggests that the CAPs are intrinsically dynamic as they fluctuate relative to the phase-based fluctuation of peak and trough.

The dynamic nature of GS is a supported by a recent study in mice^54^ by showing the instantaneous CAPs to phase lock to the ongoing fluctuations in GS. In line with their findings, Liu et al.^41^ and Zhang et al.^21^ show that in humans and monkeys, the typical GS topography, as based on phase coherence, corresponds to the CAPs extracted at the GS peak. The dynamic nature of the CAPs is further supported by the fact that they are recurring in their spatial patterns over the course of time. Moreover, Zhang et al. (2020) demonstrate that the CAPs of GS peak can further be decomposed into a subset of well-established brain networks, suggesting that the GS topography is a weighted combination of existing brain networks rather than a new unique pattern by itself. Importantly, the frequency of the CAP’s, i.e., the percentage of their occurrence, can be modulated during task states relative to the resting state—thus, GS topography can be viewed as an indicator of the transitional dynamics across brain networks.

Together, GS topography seems to be based on phase coherence and dynamic changes in fluctuating co-activation patterns (CAPs) of different networks. These findings suggest that GS topography is not a mere artifact or extra-neuronal noise but based on specific neuronal mechanisms in their network dynamics, that is, phase-based dynamics in especially the infraslow frequency range. How these neuronal mechanisms, i.e., phase coherence and dynamics of CAPs, contribute to the not yet fully clear role and function of GS for both brain and behavior remains unclear at this point.

## Dual-layer model (DLM)—integrating the two functions of GS

### Dual-layer model vs single-layer model of GS

The GS is usually traced to sources from physiological signal^10^ like respiration^31,32,53^, or projections from subcortical area like basal forebrain^41,48^. Based on these physiological sources, the function of GS is often associated with level of arousal^18,41,48,49^. The topography of GS is, previously, often seen as a consequence of the representation of these physiological sources in the brain^10^—GS topography is considered to be a mere manifestation of GS itself. This suggests what we describe as ‘single-layer model (SLM)’ of the global signal that does not differentiate GS itself and GS topography in their functions or roles. Succinctly put, such single layer model does not distinguish the role of subcortical-cortical GS in brain–body coupling including related arousal from the role of GS in structuring the dynamic topography of the cortex and its associated cognition.

However, as we mentioned above, recent findings suggest GS topography to exhibit its own intrinsic dynamics^21,54^, and is partially independent of the amplitude of GS;^22^ This suggests that GS topography cannot be viewed as static projection of GS, that is, in terms of one-to-one correspondence. Instead, extending beyond GS itself and its coupling to the bodily-based physiological signals, GS topography within the brain’s cortex itself may, in part, index the dynamic relationships between the different cortical regions and networks themselves—this is, for instance, reflected in the dynamics of the CAP during rest and task^21,29,54^. We therefore suggest that GS may have two distinct layers, (i) global fluctuation (i.e., background layer) associated with brain–body coupling and subcortical-cortical projection; and (ii) its spatiotemporal dynamics at the cortical level (i.e., surface layer), termed as GS topography previously.

The partial distinction is further supported by the distinct functions of the two layers. As explicated above, the subcortical-cortical infraslow global fluctuations^45,59^ operate in the background by mediating the level of arousal, which, in part, stems from sources in subcortical regions like the nucleus basalis meynert (NBM). While GS topography operates on the cortical surface itself by shaping the dynamic topography at its cortical surface and thereby organizes related behavior and cognition.

**Fig. 4:**
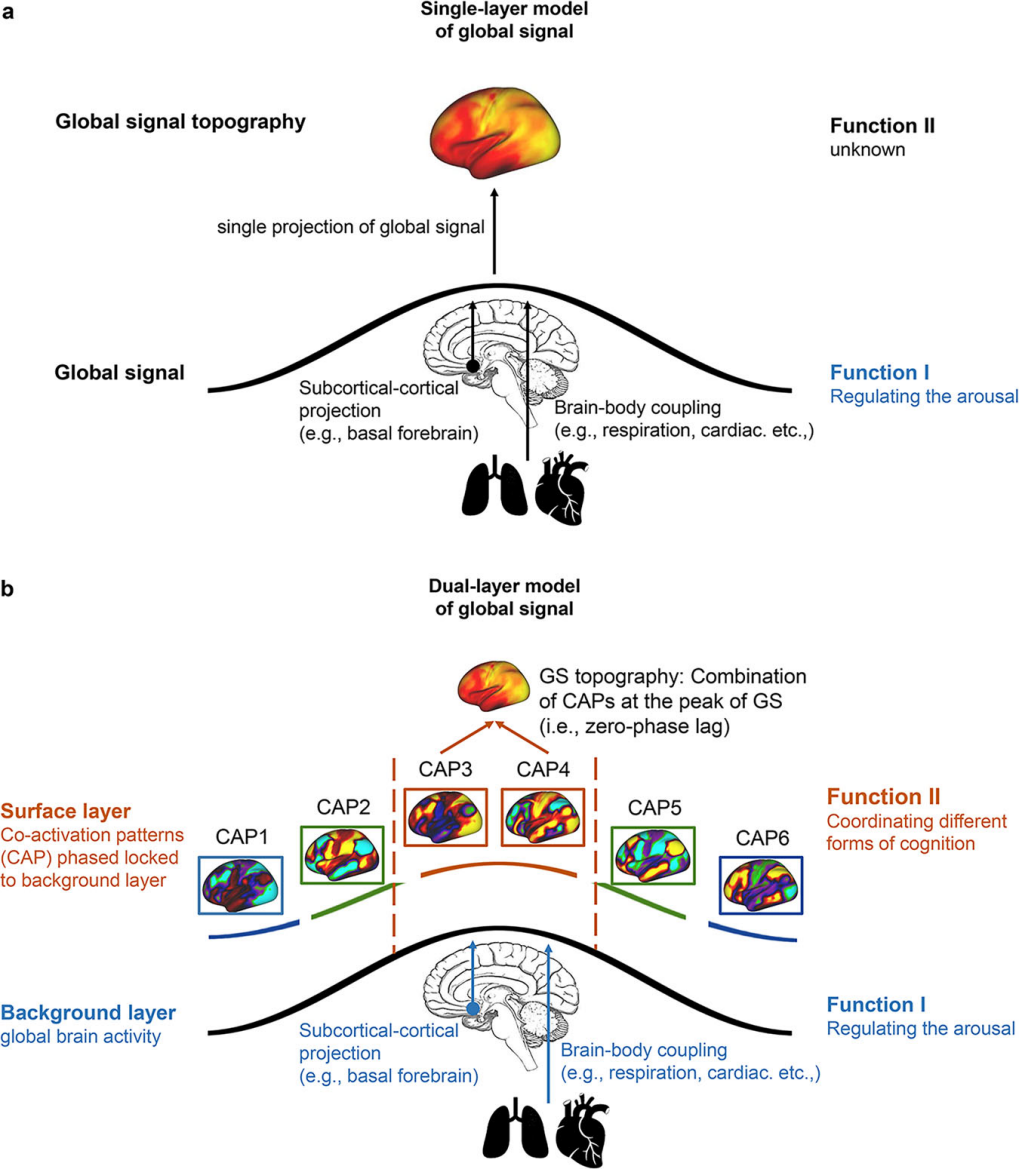
Single-layer model (SLM) vs. Dual-layer model (DLM) of GS. **a** The SLM of GS suggests that the GS stems from physiological signal like respiration and cardiac activity, or subcortical areas like basal forebrain. The cortical topography of GS is a consequence of the representation of these subcortical-cortical sources in the brain. Therefore, cortical GS topography is considered to be a mere manifestation of the subcortical-cortical GS itself with both standing in a one-to-one correspondence. **b** The DLM of GS suggests that GS is a constellation of neural activities at both a more spatially extended global background layer and a more spatially restricted surface layer featuring co-activation pattern of different networks. The background layer is the global brain activity whose neural signals, through its subcortical-cortical phase-based infraslow fluctuations, are closely coupled with the fluctuations of the bodily physiological signals like respiration, cardiac activity, and are projected from subcortical to whole brain cortical regions. That, in turn, allows for (1) regulating the level of arousal, and (2) the structuring of the dynamic topography of the cortical instantaneous brain networks/co-activation patterns (CAPs) at the surface layer as basis for coordinating different forms of cognition. Co-activation patterns CAPs; Global signal GS.

Based on these two functions including their distinct neural mechanisms, we here go beyond the single-layer view of GS, and propose a dual-layer model (DLM) of GS (Fig. 4) with background and surface layer: the background layer provides a more global infraslow fluctuation for structuring the dynamic topography of the cortical surface layer with its cortico-cortical network organization (i.e., recurring different CAPs/networks).

### Key features of the dual layer model (DLM)—distinction and dissociation of background and surface layers

Unlike in the single-layer model, the first key feature of the DLM consists in the neural distinction of more global background and more local-regional surface layers or levels of GS. Unlike in the current single layer models, the DLM considers the cortical GS topography to be partially distinct from the subcortical-cortical GS itself. If GS and GS topography were the same standing in a relationship of one-to-one correspondence, one would expect homogenous representation of global activity, e.g., GS across all regions without their topographic distinction. That is not the case, though. GS is represented in different degrees in the different networks resulting in an elaborate cortical GS topography. That is supported by recent findings in the phase-based GS topography, with strong representation in the sensorimotor network mainly occurring at the peak of GS^21,41^. In contrast, other phases of GS (like the trough, rise or fall) are tied to other networks like DMN or prefrontal network^21,54^. More importantly, even the CAPs of the GS topography itself are not a single unity; instead, they can be further decomposed into a combination of a subset of networks that co-occur at GS peak^21^. Together, these findings suggest partial neural distinction of subcortical-cortical GS and cortical GS topography. Only if we lose consciousness like in anesthesia or coma, the distinction of GS and GS topography is lost as in that case there is no distinct representation of GS in different regions/networks anymore^20^. Accordingly, a single layer model of GS may hold in the non-conscious state but not in the conscious awake state where the data suggest a dual layer model of GS.

The second key feature of DLM consists in its ability to dissociate between background and surface layers of GS. There is evidence that a changed background, i.e., alterations in subcortical-cortical GS, may co-occur with a preserved surface, i.e., intact cortico-cortical GS topography: Turchi et al.^48^ set a subcortical NBM lesion which causes global activity decrease at the contralateral hemisphere while, at the same time, the local resting state network organization is maintained. The reverse scenario, changes in the cortico-cortical surface layer co-occurring with an intact background layer is partially supported by our findings in rest-task modulation^21^. We observe that GS topography is mediated by different tasks: the frequency of task-irrelevant networks (e.g., sensorimotor network) reduces their correlation to GS, whereas the task relevant networks (e.g., visual network) increase or remain unchanged in their correlation to GS. In contrast, the basal forebrain, suggested as one of the background sources of GS^41,48^, did not show any modulation during the various tasks remaining the same throughout rest and task states^21^.

Together, these findings of partial dissociation between subcortical-cortical GS and cortico-cortical GS topography during rest and task states conforms well to the DLM. Only the DLM but not a single-layer model allows for such dynamic, i.e., flexible task-related phase-locking of recurring CAPs at the surface to an otherwise unchanged GS to basal forebrain relationship that, as providing the background, remains stable in its fluctuations across different tasks.

### Revisiting the empirical findings from the perspective of the dual layer model (DLM)

How shall we interpret the contribution of respiration to GS, as the major confound, under the view of DLM? This is not fully clear yet. One possibility is that as a global effect, the contribution of respiration to GS may, in part, reflect the background layer. The key role of the background layer of GS seems to consist in the coupling or aligning its neural signals to the body’s physiological signals (like respiration) through their shared infraslow fluctuations—psycho-physiologically, such brain–body coupling is manifest in the level of arousal (as the main function of GS as background layer).

At the same time, the global infraslow fluctuations of the GS background layer with its brain–body coupling also shape the dynamic topography of the brain’s cortical surface layer—the latter’s regional activity may thereby be (indirectly) linked to the body’s physiological signals—this is supported by the many studies that associate physiological signals like respiration and cardiac (and even stomach) with the cortical activity in various regions^31,32,96,97^. In that case, one would expect that these physiological interoceptive inputs to single regions interact with their exteroceptive inputs during task states: if the exteroceptive inputs are stronger, they should elicit activity changes while transiently decreasing their activity related to the physiological interoceptive sources. This is indeed suggested by the findings of Zhang et al.^21^.

However, the exact relationship of GS and respiration remains to be explored. This includes future questions whether the relationship between GS and respiration can be traced to the sensorimotor network (as related to the activation of the diaphragm as key muscle for respiration) coordinated by GS, how the respiration as an internally-oriented processing is mediated by more externally-oriented tasks under the coordinating influence of GS. The findings of uniformly decreased GS throughout the whole brain and its relation with the level of arousal^20^ may shed some important light on the key role of the GS background layer for the dynamic topography at the cortical surface and associated consciousness: loss of the background layer’s infraslow fluctuations dedifferentiates the cortico-cortical dynamic topography which, becoming homogenous, renders its incapable to react to intero- and exteroceptive inputs in a differentiated way. Both types of inputs can consequently no longer be perceived in a differentiated way as manifest in the loss of consciousness. Future task studies of GS in both awake and unconscious states are warranted to support such assumption.

How can we interpret the empirical findings from the psychiatric disorders in the view of DLM? The DLM provides an approach to investigate in the future about whether psychiatric disorders are (1). primarily disorders of the background layer of GS, or (2) disorders of its surface layer (or both layers). The findings in schizophrenia suggest involvement of both layers as the results show that increased GS co-occurs with altered GS topography^19,81^. While bipolar disorder, in contrast, seems to exhibit primarily changes in GS topography, i.e., the surface layer, with the background layer remaining ‘normal’^22^. The same seems to be the case in MDD where GS exhibits abnormal shifts in its topography towards the DMN^89^. The fact that changes in GS and GS topography lead to distinct psychopathological symptoms in these disorders (like bipolar disorder and schizophrenia), suggest a differential role of the two layers of GS in shaping our behavior and cognition, namely in coordinating the different sensory, motor, affective, and cognitive functions^86^.

### How can we test the dual-layer model?

How can we measure and disentangle the two layers, background and surface layer of GS? The DLM suggests that there are two components in the observed ‘GS’, that is, a global brain activity extending more or less across the whole brain (background layer) and, phase-locked to that, a dynamic change in network constellations, i.e., phase-locked CAPs (surface layer). However, based on the measurement of GS, that is, the overall activity across all cortical regions, it remains unknown whether the GS, as we measure it, is a mixture of these two layers, or, alternatively only a summation of the surface network itself without any impact of the truly global background layer. In the latter case, the ongoing phase fluctuations of GS would be exclusively determined by the summation of the overall activity of the cortico-cortical CAPs, rather than by a truly global subcortical-cortical brain activity (as that remains partially independent of its topographical representation in the CAPs).

How can we decide this issue? Methodologically, one approach is to test if the background layer (i.e., the global activity and its globally extending fluctuations) still holds after removing the surface layer (i.e., CAPs). After regressing the activity of CAPs, the residual of GS may still display the same temporal structure—this will support the partially distinct existence of the subcortical-cortical background layer as distinct from the cortico-cortical surface layer. In that case, one would expect that the brain–body coupling of neural and physiological signals through the GS infraslow fluctuations remains intact while, due to lack of cortical dynamic topography, there is no differentiation anymore on the cortico-cortical level of input processing. That may, as we assume, be the case in sedation or those states like N2/3 sleep when one is an unconscious state but can still awake in the presence of a strong exteroceptive input (like a loud tone or noise during sleep); this is different for instance in full surgical anesthesia, as long as one is on the anesthetic drugs, where the background layer itself is altered, i.e., the subcortical-cortical infraslow fluctuations of background GS^20^. Alternatively, another approach is to check the residuals after regressing the background layer by, for example, regressing the activity from basal forebrain which is supposed to provide one source of global activity: if the spatial pattern of the residual becomes more similar to the standard CAP, it may also support the existence of DLM in GS. The separation of background and surface layer of GS might also be tested by manipulating the level of arousal as well as by investigating the interaction between cognitive dynamics and arousal. For example, one could alter the level of arousal (i.e., background layer) by pharmacological interventions^62^, transition from sleep to awake^98,99^, physical exercise^100^, or blocking the activity of certain neuronal population that are related to the ascending arousal system^101,102^. In those instances, one would expect that the spatiotemporal patterns on the cortical level (i.e., surface layer) with their dynamic topography are somewhat preserved – this would indicate the partial independence or dissociation of the surface layer of GS topography from the background payer of GS.

## Conclusion

Global activity and its impact on local-regional activity are common phenomena of complex systems in the natural world as documented for instance in climate change and economy. Analogously, the brain too exhibits ‘global’ activity which, as measured with the global signal (GS), strongly shapes its more local-regional activity. GS is often discarded as mere noise that is to be eliminated from the data. However, recent evidence accumulates to a different view of GS, namely that it takes on an important physiological role and function for brain–body coupling, the brain itself, and associated behavior and cognition.

We here review recent findings about electrophysiological basis, and two types of function of GS. These include (i) mediation of the level of arousal through subcortical-cortical infraslow coupling of neural and physiological signals (function I); and (ii) coordination of the different forms of cognition through organizing a phase-based dynamic topography of cortico-cortical interactions (function II). Based on these two functions, we propose a Dual-layer model (DLM) of GS where global infraslow fluctuations provide the neural background layer for a more localized activity at the cortico-cortical surface layers. We conclude that such Dual-layer model of GS extends GS beyond noise by allowing for a more comprehensive view of its role in both brain–body coupling, e.g., arousal, and dynamic topography of the cortex organizing our cognition.

### Data for reference

This is a review article. All the data and sources mentioned in this paper were cited and stated in the corresponding positions.
