# Author Correction: Beyond noise to function: reframing the global brain activity and its dynamic topography

**DOI:** 10.1038/s42003-022-04379-5

**Published:** 2022-12-21

**Authors:** Jianfeng Zhang, Georg Northoff

**Affiliations:** 1 Center for Brain Disorders and Cognitive Sciences, Shenzhen University, Shenzhen, China; 2 School of Psychology, Shenzhen University, Shenzhen, China; 3 Mental Health Center, Zhejiang University School of Medicine, Hangzhou, China; 4 Institute of Mental Health Research, University of Ottawa, Ottawa, Canada; 5 Center for Cognition and Brain Disorders, Hangzhou Normal University, Hangzhou, China

**Keywords:** Consciousness, Dynamical systems, Cognitive neuroscience

Correction to: *Communications Biology* 10.1038/s42003-022-04297-6, published online 08 December 2022.

The original version of this Article contained an error in which the images used for figures 2 and 3 were swapped. All images and panels used for figure 2 in the original version should be used for figure 3 in the new version. Conversely, all images and panels for figure 3 in the original version, should be used for figure 2 in the new version.

This error has now been corrected in the PDF and HTML versions of the Article.

